# Polymeric Materials Reinforced with Multiwall Carbon Nanotubes: A Constitutive Material Model

**DOI:** 10.3390/ma6072873

**Published:** 2013-07-16

**Authors:** René K. Córdova, Alex Elías-Zúñiga, Luis E. Elizalde, Héctor R. Siller, José Antonio Sánchez, Ciro A. Rodríguez, Wendy Ortega

**Affiliations:** 1 Tecnológico de Monterrey, Av. Eugenio Garza Sada 2501 Sur Monterrey CP64849, Mexico; E-Mails: rene.cordova@itesm.mx (R.K.C.); hector.siller@itesm.mx (H.R.S.); asanfer@itesm.mx (J.A.S.); ciro.rodriguez@itesm.mx (C.A.R.); wlortega@itesm.mx (W.O.); 2 Centro de Investigación en Química Aplicada, Blvd. Enrique Reyna Hermosillo 140 Saltillo, Coahuila CP25250, Mexico; E-Mail: elizalde@ciqa.mx

**Keywords:** reinforced polymers, carbon nanotubes, softening effect, constitutive model

## Abstract

In this paper we have modified an existing material model introduced by Cantournet and co-workers to take into account softening and residual strain effects observed in polymeric materials reinforced with carbon nanotubes when subjected to loading and unloading cycles. In order to assess the accuracy of the modified material model, we have compared theoretical predictions with uniaxial extension experimental data obtained from reinforced polymeric material samples. It is shown that the proposed model follows experimental data well as its maximum errors attained are lower than 2.67%, 3.66%, 7.11% and 6.20% for brominated isobutylene and paramethylstyrene copolymer reinforced with multiwall carbon nanotubes (BIMSM-MWCNT), reinforced natural rubber (NR-MWCNT), polybutadiene-carbon black (PB-CB), and PC/ABS reinforced with single-wall carbon nanotubes (SWCNT), respectively.

## 1. Introduction

Over the last few decades, the Mullins effect has been studied to characterize stress-softening effects observed in elastomeric materials. However, a few studies have been conducted on modeling the mechanical behavior of nanocomposite elastomers reinforced with carbon nanotubes in which Mullins and residual strain effects are considered. The characterization of the mechanical behavior of reinforced nanocomposite elastomers is of great importance in many engineering applications since the accurate predictions of its mechanical response are essential to design and manufacture of new products based on nanotechnology developments. For instance, the combination of low volume fraction of carbon nanotubes (CNTs) suggests that CNTs are ideal candidates for high performance polymer composites [1] since single-wall carbon nanotubes (SWCNTs) have shown exceptional mechanical properties such as an increase in the values of the Young modulus and the maximum material strength [2,3,4].

To model the behavior of a carbon nanotube-reinforced polymeric materials (CNRPs), Dikshit and co-workers [5] applied constitutive equations developed by Mulliken and Boyce for amorphous materials [6] and hypothesized that a polymeric material reinforced with SWCNT can be considered as an heterogeneous material represented with component phases having different constitutive behaviors. Cantournet and co-workers [7] proposed a hyperelastic constitutive model for a MWCNTs-reinforced elastomer to describe the material behavior, assuming that the strain energy of the elastomeric material can be computed by using the Arruda–Boyce model which considers the material to be isotropic and isochoric [8,9], while the anisotropic strain energy function of the MWCNT is “isotropized” by considering the average orientations of the MWCNTs given with respect to the principal stretch directions, *i.e.*, taking the azimuthal angle of 55°, and its corresponding magnitude is subsequently computed by using the rule of mixtures. However, these models are mainly focused on predicting the virgin loading curve of cyclic tension tests. Therefore, the aim of this article is to develop a model that will take into account Mullins and residual strain effects since these phenomena occur in materials that have many engineering applications [10,11,12,13,14,15,16,17,18,19,20]. Since the model is based on the material strain energy density, it could be feasible to explore its application to predict the mechanical response behavior of composite materials with a glassy polymer matrix if a thermoplastic equivalent constitutive material model is used to describe the glassy material behavior. At the end of the paper, we will show that our proposed model will suffice to model with good precision only the elastic region of a PC/ABS reinforced with SWCNTs and carbon black (CB) particles; this is possible by considering that the average orientation of these fillers is aligned with respect to the principal stretch directions and, therefore, we can assume that the mechanical response behavior of a polymeric material subjected to uniaxial extension and reinforced with these fillers can be considered to be an “isotropized” material [7]. Before we proceed with the derivation of a material model that will consider stress-softening and residual strain effects in polymeric materials reinforced with carbon nanotubes, we shall first begin by briefly reviewing some basic concepts of finite elasticity.

## 2. Basic Concepts

In this section, we review some kinematic relationships for finite deformations of incompressible, hyperelastic materials. First, let us consider a material particle at the place **X** = *X_k_***e***_k_* in an initially undeformed reference configuration of a body. When subjected to a prescribed deformation, the particle at **X** moves to the place **X** = *x_k_***e***_k_* in the current configuration of the body in a common rectangular Cartesian frame *φ* with the origin at *O* and an orthonormal basis **e***_k_*. An isochoric deformation is described by:

*x*_1_ = *λ*_1_*X*_1_; *x*_2_ = *λ*_2_*X*_2_ ; *x*_3_ = *λ*_3_*X*_3_ (1)

in which λ_i_, *i* = 1, 2 and 3, denote the principal stretches in *φ*. The Cauchy–Green deformation tensor **B** = **FF**^T^ has the form:

(2) $$B=\lambda_{1}^{2}e_{11}+\lambda_{2}^{2}e_{22}+\lambda_{3}^{2}e_{33}$$

where **e**_*jk*_ = **e**_*j*_ ⊗ **e**_*k*_, **e**_*i*_, are associated orthonormal principal directions; and **F** is the usual deformation gradient. The magnitude of the strain at a material point **X**, also called the *strain intensity* and denoted by *m*, is defined by 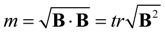 where *tr* denotes the trace operation. In the undeformed state **F** = **1**, and the strain intensity $m=\sqrt{3}$; otherwise $m>\sqrt{3}$ for isochoric deformations [21].

The principal invariants *I*_k_ of **B** are defined by:

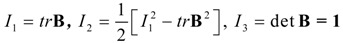
(3)

Thus, the magnitude *m* of **B** as a function of the invariants is given by:

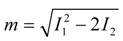
(4)
here $m=\sqrt{3}$ when and only when *λ* = 1, the unstrained deformation state. Also, for all deformations of an incompressible material, *I*_3_ = 1.

To model the material stress-softening behavior, we shall assume that the microstructural material damage is characterized by a certain isotropic and non-monotonous increasing function *F*(*m*;*M*) that depends on the material strain intensity *m* and satisfies the conditions:

0 < *F* (*m*; *M*) < 1; *F* (*M*; *M*) = 1 (5)

where *M* represents the maximum previous strain intensity at the point at which the material is unloaded from its virgin material path. The softening function *F*(*m*;*M*) is determined by a constitutive equation that describes the evolution of micro-structural changes that begin immediately upon deformation from the natural, undistorted state of the virgin material. We assume that *F*(*m;M*) is a positive non-monotones-increasing function of the strain intensity on the interval $m\in (\sqrt{3,}M)$. If we let *M* be the amount of stretch at the point at which the material is unloaded and fix the maximum previous strain intensity energy at *m = M*, then the stress-softening material response for subsequent unloading and reloading again from an unstrained state, or from any other elastic point for which *m = M* is defined by the following time-independent constitutive equation

**τ** = *F* (*m*; *M*) **T** (6)

where **τ** denotes the Cauchy stress tensor in the stress-softened material and **T** denotes the Cauchy stress tensor during loading of the virgin material.

Based on the non-monotonous behavior of reinforced rubber-like materials [21], here we assume that the softening function has the form

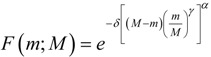
(7)
where *δ* is a positive softening material parameter; *α* and *γ* are positive scaling constants chosen to best fit experimental data for a given rubber-like material. Substitution of Equation (7) into Equation (6), gives:

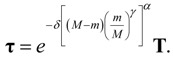
(8)

Notice that for our constitutive material model given by Equation (8), the ratios of the nontrivial physical stress components *T*_ij_ in the virgin material to the corresponding nontrivial physical components *τ*_ij_ in the stress-softening material, for a given deformation state, are determined by the inverse of the softening function alone:

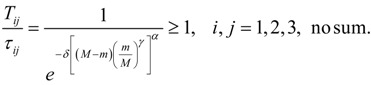
(9)

**Figure 1:**
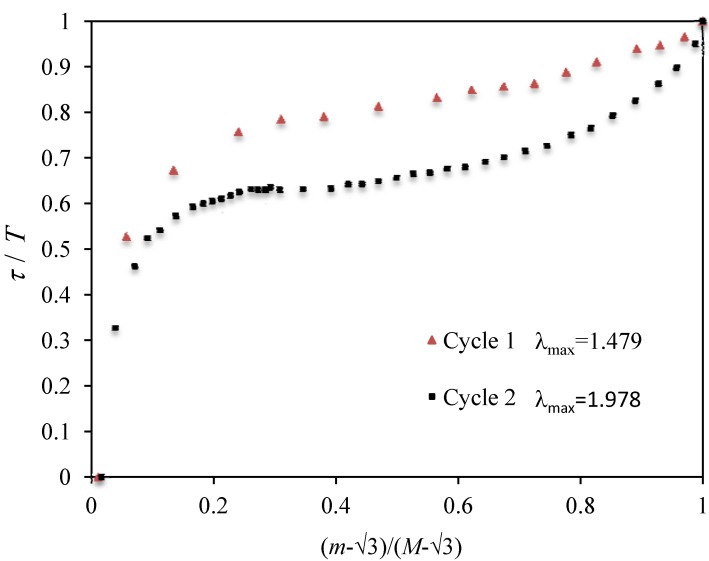
Normalized experimental stress data for the first two loading–unloading cycles of uniaxial tension tests performed in BIMSM-MWCNT (12.2%) composite material plotted against the normalized strain intensity ratio. Experimental data adapted from [7].

The simple rule given by Equation (9) provides an expression to determine the softening parameters from experimental data as shown in Figure 1, in which the first two loading–unloading cycles are considered to compute the corresponding values of *α*, *δ*, and *γ*.

A similar procedure was followed by Gurtin and Francis [22] by considering a one-dimensional softening damage function to describe internal damage in highly filled solid propellants subjected to simple uniaxial tension tests; however, their results did not collapse to a single curve for all values of *λ*_max_. Also, please note that various hypothetical damage functions, bearing properties similar to Equation (7), have been proposed in the literature. However, these have monotone-like behavior. See, for instance, [3,21,23,24,25,26] and references cited therein.

## 3. “Isotropized” Model

In this section, we review the main features of the Cantournet *et al.* model [7] and show how the strain energy densities of the polymeric matrix and the volumetric fraction of carbon nanotubes can be computed.

By following the results found in [7], the strain energy density *U_c_*, of the MWCNT composite elastomer material can be found by adding the strain energy density of the elastomeric part *U_e_*, to the strain energy density associated with the MWCNTs, *U_MWCNT_*. It was also assumed that the strain energy density of the MWCNT’s is giving by the following rule:

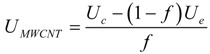
(10)
where *f* is the volumetric fiber fraction. The elastomeric strain energy density *U_e_* is also assumed to be given by the compressible version of the eight chain model:

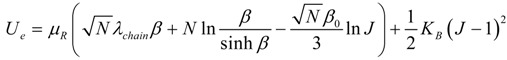
(11)
where *N* is the chain number of links, 
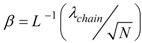
, 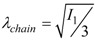, 
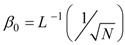
, 
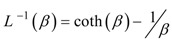
, 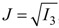 and *K_B_* is the material bulk modulus, *Ը*^−1^ is the inverse of the Langevin function, and *I*_1_ and *I*_3_ are the first and third invariants of the left Cauchy–Green deformation tensor. Thus, the Cauchy stress tensor due to the elastomeric matrix **T**_e_, can be obtained by the following expression:

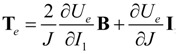
(12)
which can be rewritten as

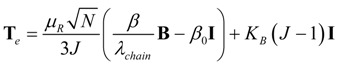
(13)
where **B** is the left Cauchy–Green deformation tensor and *μ_R_* is the material shear Young modulus. Note that the second term becomes zero if the material is incompressible, *i.e.*, *J* = 1.

Cantournet and co-workers make the assumption that the MWCNT strain energy, *U_MWCNT_*, can be expressed by an “isotropized” relation described by:

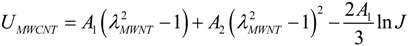
(14)
or in equivalent form by

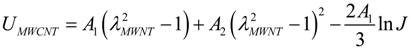
(15)
where *A*_1_ and *A*_2_ are the “isotropized” parameters fitted from the *U_MWCNT_* *vs.* 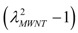 data plots by using uniaxial experimental data.

Since the Cantournet model states that the composite strain energy function is simply the sum of the contributions from the elastomer material and from the MWCNTs, the Cauchy stress constitutive equation of the composite material can thus be written as:

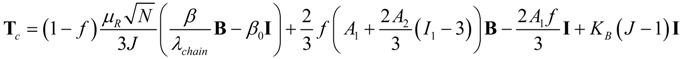
(16)

It is important to mention that, in general, the Cantournet model [7] fits loading virgin paths for lower volumetric MWCNT fraction and for relative small deformations well, but tends to overestimate experimental data for higher volumetric fractions of MWCNT at large deformations [7].

## 4. Inclusion of Residual Strain Effects

This section describes a method to predict analytically the permanent set phenomenon of rubber-like materials. To take into account residual deformations, we follow the Holzapfel *et al.* model [27] and assume that the strain energy function *W* has the form

*W* = *U_e_* (*λ*_1_, *λ*_2_, *λ*_3_) + *Ŵ_rs_* (*λ*_1_, *λ*_2_, *λ*_3_, *ξ*_1_, *ξ*_2_, *ξ*_3_) ‒ *p*(*J* ‒ 1) (17)

where the function *U_e_* represents the strain energy density of the composite material associated with the primary loading path; *Ŵ_rs_* is the strain energy density function related to the material damage mechanism during unloading conditions; *ξ_a_*, a = 1, 2, 3 represent the discontinuous damage variables; *p* is an arbitrary hydrostatic pressure; and *J* = *λ*_1_*λ*_2_*λ*_3_ = 1 due to the incompressibility condition.

In accordance with the pseudo-elasticity theory introduced in [28], the damage energy density function must satisfy the following relationship:

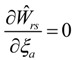
(18)

By using the concepts of the pseudo-elasticity theory, it is possible to show that the discontinuous damage variables *ξ_a_* are given as

(19) $$\xi_{a}=\frac{1}{C}(\lambda_{a}^{n}-\lambda_{a\;\text{max}}^{n})$$

where *C* represents a material constant related to the damage mechanism, and *λ_a_*_max_ are the maximum values of the principal stretches at which unloading on the primary loading path begins. In accordance with the pseudo-elasticity theory, on the primary loading path *ξ_a_* must be inactive, while on the unloading path, *ξ_a_* has the value given by Equation (19). In this case, the damage energy density function which depends on the positive scaling parameter *n* can be expressed as [29]

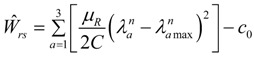
(20)

Here, *c*_0_ is an integration constant, and *n* is a positive scaling parameter that in general takes the value of one for the proposed material model. Thus, the supplementary stress components $T_{rs_{a}}$ , in the principal directions, can be computed as the derivative of the damage energy density with respect to the principal stretches:

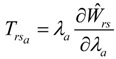
(21)
where *∂Ŵ_rs_*/*∂λ_a_* is given as

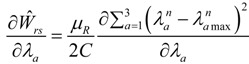
(22)

Substitution of Equation (22) into Equation (21), yields the expression to compute the residual stress components $T_{rs_{a}}$:

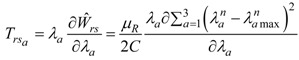
(23)

Then, the corresponding constitutive equation that characterizes the material behavior during the unloading path that takes into account not only the non-monotonous softening effects but also residual strains, can be described by

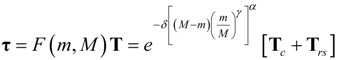
(24)
where **τ** denotes the Cauchy stress of the stress-softening material, and **T**_c_ is given by Equation (16).

Finally, and by recalling Equation (23), the stress–stretch constitutive material model that predicts softening and residual strain effects for polymeric-like reinforced materials, for a three-dimensional deformation state, is given by

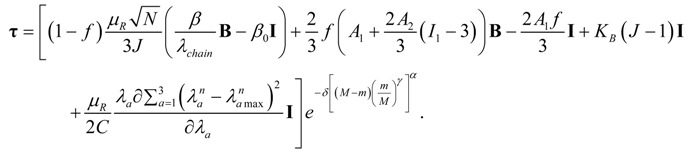
(25)

## 5. Comparison of Theoretical Predictions with Experimental Data

In this section, we compare uniaxial experimental data with theoretical predictions provided by Equation (25). The experimental data are taken from different elastomeric materials mainly reinforced with carbon nanotubes: BIMSM-MWCNT [7], natural rubber-MWCNT [30], reinforced polybutadyene with carbon black particles PB-CB and a thermoplastic polymer blend PC/ABS reinforced with SWCNT.

First, we compare experimental data of the elastomeric material called BIMSM-MWCNT [7]. Here, we choose the parameter values of *α* = 1/2, and *γ* = 1 in Equation (25) and then we fit the value of the softening parameter *δ* and the chain number of links *N* = 30 by following a procedure similar to the one described in [7]. The shear modulus *μ*_R_, and the “isotropized” parameters, *A*_1_ and *A*_2_, for each volumetric fraction of MWCNT are listed in Table 1.

**Table 1:** Material parameter values of *A*_1_, *A*_2_, and *μ*_R_ for different weight volume fractions of MWCNTs.

Material parameter values	2.6% w	6.1% w	8.8% w	12.2% w
*A*_1_ (MPa)	6.61	8.39	8.01	12.4
*A*_2_ (MPa)	2.31	3.16	2.24	3.33
*μ*_R_(MPa)	0.45	0.43	0.43	0.44

**Figure 2:**
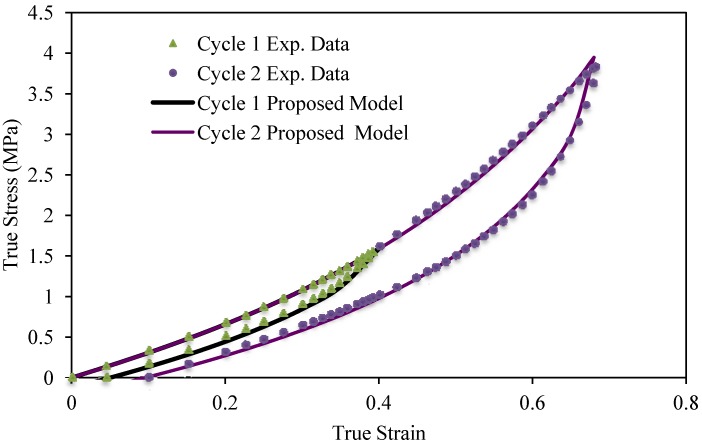
Theoretical predictions obtained from the proposed material model given by Equation (25) compared with experimental data for 12.2% of MWCNT. The estimated material parameter values are *δ* = 0.49, and *C* = 4 MPa.

The damage parameter values for each volumetric fraction of MWCNTs are also summarized in Table 2. The residual strain parameters, shown in Table 2, are fitted by substituting Equation (23) into Equation (24). We next use Equation (25) and compute theoretical predictions by considering a volumetric fraction of 12.2% of MWCNT. As we can see from Figure 2, there is good agreement between experimental data and theoretical predictions mainly at lower stretch values.

**Table 2:** Damage parameter values for different weight volume fractions of MWCNTs.

Weight fraction	0.0%	2.6%	6.1%	8.8%	12.2%
*δ*	0.05	0.08	0.10	0.13	0.36
*γ*	1	1	1	1	1
*C* (MPa)	6.00	8.00	6.40	5.88	1.80

**Figure 3:**
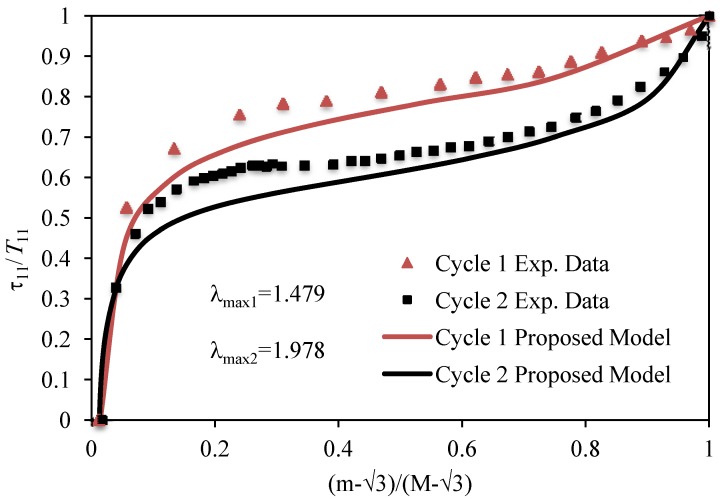
Comparison of theoretical predictions computed from Equations (16) and (25) with experimental data collected from a 12.2% w fraction of MWCNT-reinforced elastomer by plotting the normalized stress ratio *τ*_11_/*T*_11_ *versus* the normalized strain intensity ratio (*m* – √3)/(*M* – √3)

We now use Equation (8) and Equation (25) to compare this experimental data with theoretical predictions by plotting the ratio of the stress-softening stress, *τ*_11_, and the virgin stress, *T*_11_, *versus* the strain intensity ratio of $\left(m-\sqrt{3})/(M-\sqrt{3}\right)$ for the first two cycles, as illustrated in Figure 3. As we can see from Figure 3, theoretical predictions follow experimental data well. Similar plots can be obtained by considering different volumetric fractions of MWCNT. Table 3 summarizes the root-mean-square error (RMSE) obtained in the first loading and unloading curves, respectively.

**Table 3:** RMSE at different percents of weight volume fractions of MWCNTs for loading and the first unloading paths.

MWCNT,%	RMSE Loading path	RMSE First unloading path
2.6	0.039	0.042
6.1	0.048	0.050
8.8	0.053	0.054
12.2	0.054	0.075

The maximum predicted error, *e*, for the 12.2% wt fraction is 2.67% for both loading and unloading curves. The corresponding RMSE is 0.048 for cycle 2 of the experimental data shown in Figure 2. Notice that for high volumetric fractions of MWCNT, the magnitude of the RMSE between experimental data and theoretical predictions has a small increasing value. Therefore, we can conclude that the modified Cantournet model tends to follow data even for high volumetric fractions of MWCNT in the BIMSM-MWCNT-reinforced polymer well. The corresponding RMSE values at different volumetric factions of MWCNT’s for the first loading–unloading material cycle are summarized in Table 3.

**Figure 4:**
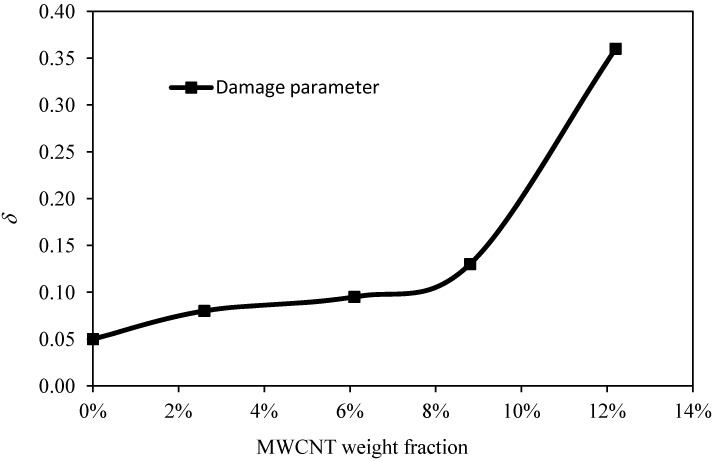
Softening parameter behavior, *δ*, as a function of the material weight fraction of MWCNT.

In Figure 4, we have plotted the variation of the softening parameter *δ* *versus* the weight fraction of MWCNTs added to the polymeric matrix of the BIMSM polymer. As we can see from Figure 4, when the weight fraction increases, the softening parameter *δ* also tends to increase. This plot can be used as a reference curve to predict the value of *δ* at different weight fractions of MWCNTs.

**Figure 5:**
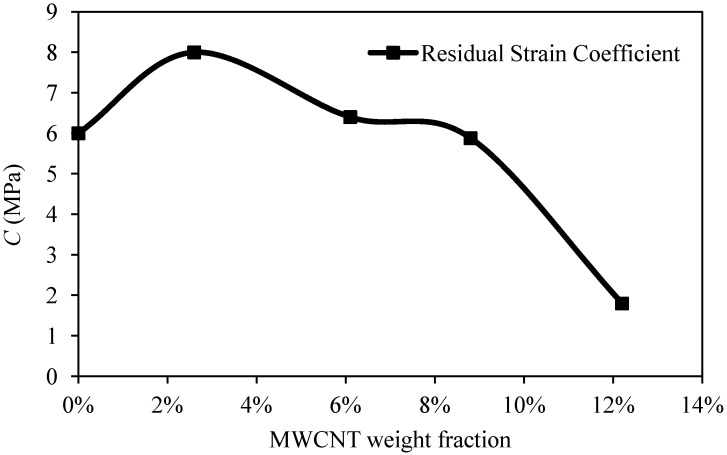
Residual strain material parameter values, *C*, plotted as a function of the weight fraction of MWCNT.

Figure 5 shows the variation of the residual strain material parameter *C versus* the volumetric fractions of MWCNTs. Note that the values of *C* tend to decrease at higher volumetric fractions of MWCNTs; also, its curve exhibits a non-monotonous behavior.

**Figure 6:**
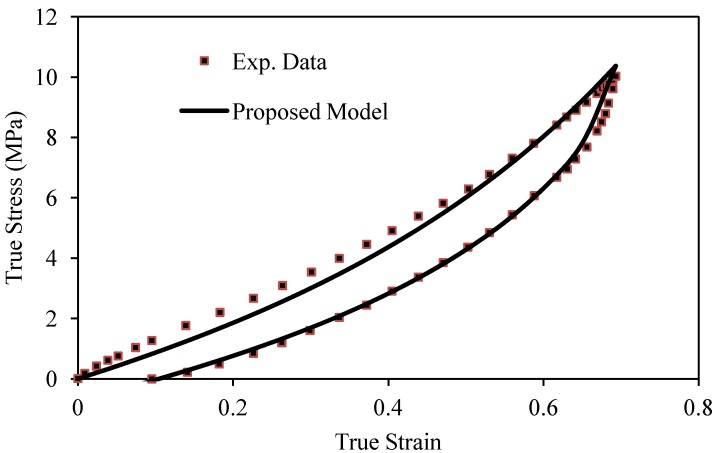
Comparison of theoretical predictions obtained from the proposed model given by Equation (25) with experimental data for 1 phr NR-SWCNT. Here the material parameter values are *δ* = 0.3 and *C* = 1.0 MPa. Experimental data adapted from [30].

We next use uniaxial extension experimental data obtained from Nah *et al.* [30] for reinforced natural rubber to assess the accuracy of our modified Cantournet *et al.* model given by Equation (25) by considering the parameter values of *μ*_R_ =0.75 MPa, *δ* = 0.3, *C =* 1.0 MPa, *n* = 1, *γ* = 1, *α* = 0.5, *A*_1_ = 137 MPa, *A*_2_ = 10.8 MPa and *N* = 30. As we can see from Figure 6, theoretical predictions tend to underestimate experimental data mainly at lower strain values.

However, the qualitative and quantitative behavior exhibited by experimental data are accurately predicted from our proposed material model given by Equation (25). In this case, the computed RMSE values are 0.392 and 0.144 for the loading and unloading paths, respectively. The maximum error attained is 3.66% computed at the maximum stretch value shown in Figure 6.

**Figure 7:**
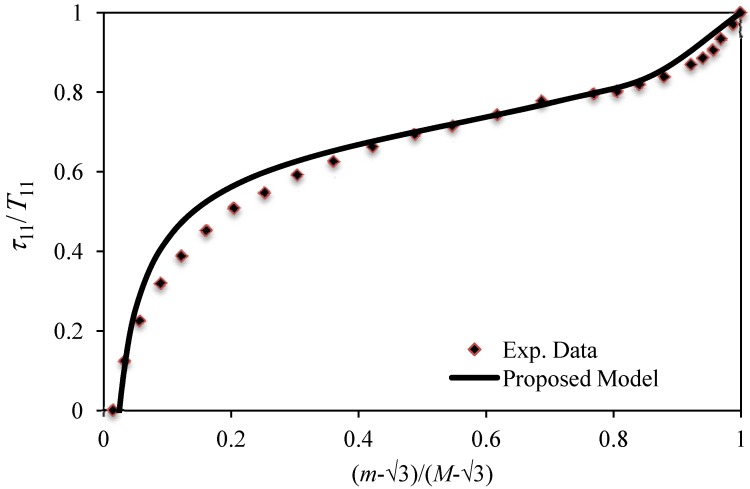
Comparison of theoretical predictions with respect to experimental data for 1 phr NR-MWCNT. Experimental data adapted from [30].

Figure 7 shows theoretical predictions of the normalized stress ratio *τ*_11_/*T*_11_ plotted against the normalized strain intensity ratio $\left(m-\sqrt{3})/(M-\sqrt{3}\right)$ by using Equations (16) and (25). As we can see from Figure 7, theoretical predictions follow experimental data well.

**Figure 8:**
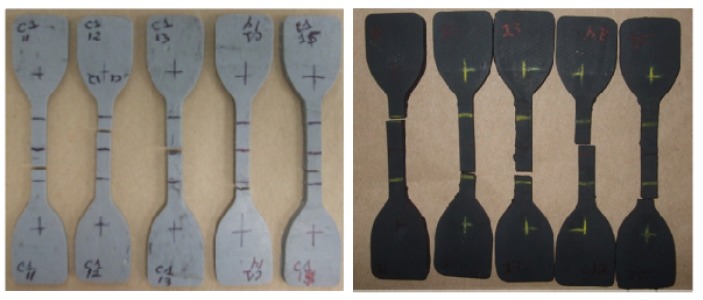
Specimens of PBR and PBR-CB subjected to uniaxial cyclic tension tests, according to ASTM D412 Rev A, standard die C.

**Figure 9:**
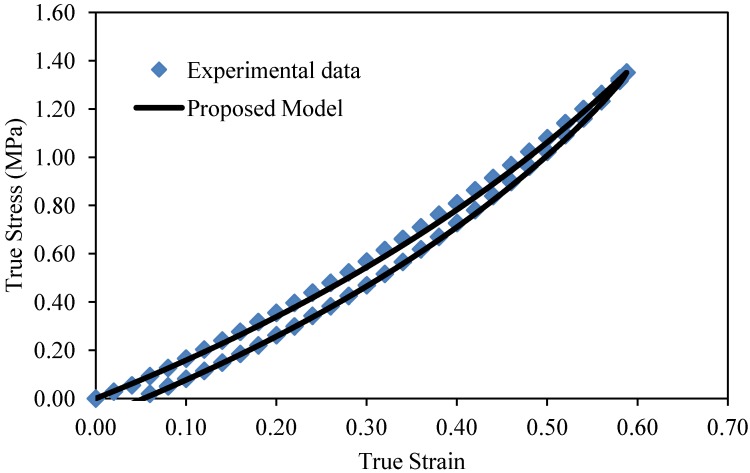
Comparison of experimental data with respect to stress-softened predictions obtained from Equation (25) for 1 phr of CB-reinforced elastomer.

We now use uniaxial extension experimental data obtained from samples of PBR-CB elastomer according to ASTM D412 Rev A, standard die C collected by using an universal testing machine MTS insight 2 with a load cell capacity of 2 kN. Some of the samples tested are shown in Figure 8. These samples are commercial blends described in Table 4. Comparison of experimental data obtained at the constant strain rate of 0.3 s^−1^ and theoretical predictions is shown in Figure 9 for the first loading and unloading cycle. The estimated parameter values are *A*_1_ = 6.213 MPa, *A*_2_ = −0.2549 MPa, *µ*_R_ = 0.45 MPa, *δ* = 0.05, *N* = 30, *C* = 5.5 MPa, *n* = 1, *α* = ½, and *γ* = 1.

**Table 4:** Composition for the two samples of polybutadiene (the unit is phr).

Chemical composition	Sample 1P (phr)	Sample 2 (phr)
Polybutadiene	100	100
CB	0	7
ZnO	5	5
S/A	1	1
MBTS	1	1
Sulfur	2	2

As we can see from Figure 9, there is good agreement between theoretical predictions and collected experimental data. In this case, the RMSE has the values of 0.0401 and 0.0107 for loading and unloading curves, as shown in Table 5. The maximum error value is 7.11% computed at the stretch value of *λ*_max_ = 1.8.

**Table 5:** Predicted median square (MSE) and quadratic (RMSE) error values for a PBR-CB composite material.

Computed error values	Loading	Unloading
MSE	0.0016	0.0001
RMSE	0.0401	0.0107
Maximum Error	7.11%	1.94%

**Figure 10:**
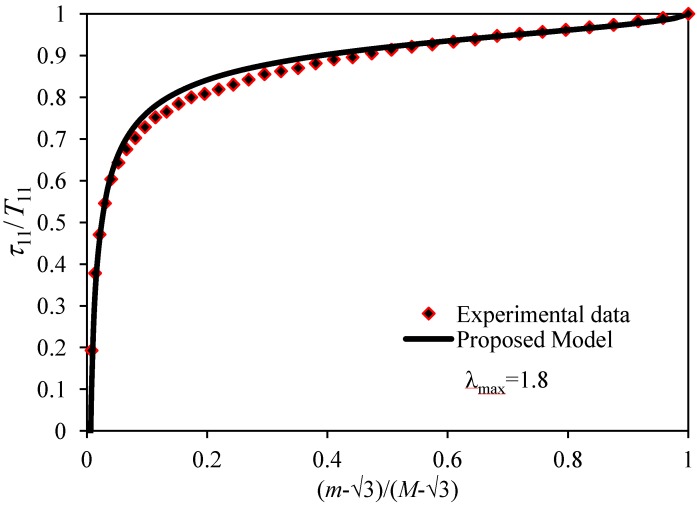
Comparison of experimental data and the normalized predicted material stresses *τ*_11_/*T*_11_ for the first cycle of loading and unloading for the PBR-CB composite material.

Figure 10 shows the ratio of the softening stress *τ*_11_, and the virgin stress *T*_11_, plotted *versus* the normalized strain ratio $\left(m-\sqrt{3})/(M-\sqrt{3}\right)$. Notice that our proposed non-monotonous softening model, along with the inclusion of residual strains, predicts well the behavior of the PBR-CB composite material.

**Figure 11:**
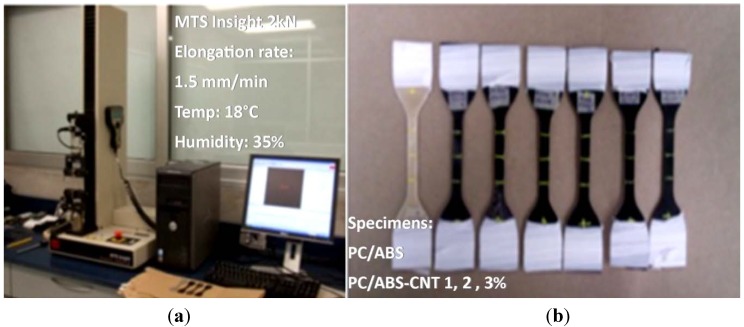
Experimental setup for PC/ABS composites. (**a**) Universal testing machine MTS insight 2; and (**b**) PC/ABS-SWCNT specimen dumbbell shape Die C according to the ASTM D638 type IV.

**Figure 12:**
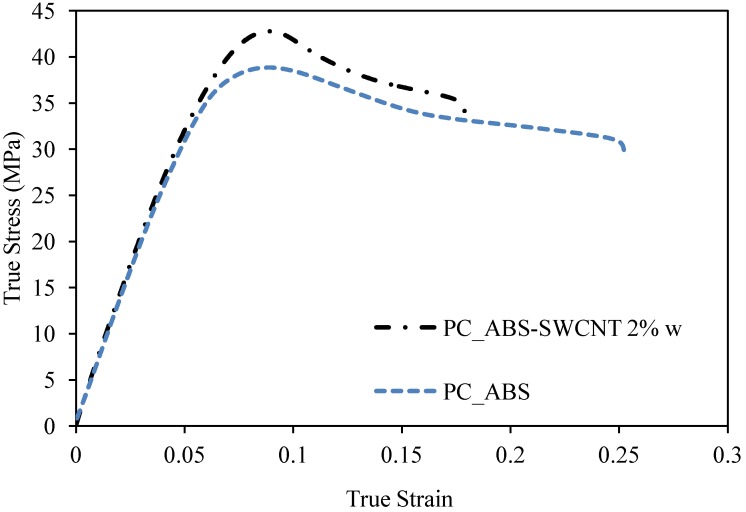
Collected uniaxial experimental data for PC/ABS-SWCNT composite material samples with 2% weight fraction of SWCNT at the strain rate of 0.001 s^−1^.

We next characterize samples of PC/ABS reinforced with SWCNTs with 2% and 3% of weight volume fraction. In this case, we followed the ASTM-D638 type IV, and used the universal testing machine, MTS Insight 2 from MTS Insight^®^ electromechanical testing system with a load cell capacity of 2 kN to perform two different tests: (a) uniaxial tension test to failure; and (b) uniaxial cyclic tests with a constant incremental elongation. Figure 11 shows the experimental set up and the material samples. We performed cyclic tests for each SWCNT fraction at the strain rate of 0.001 s^−1^. Figure 12 shows the results for uniaxial cyclic test in which there is a slight increment of the strength value when the material is reinforced with 2% of SWCNT.

We next use our material constitutive model given by Equation (25) to predict the mechanical behavior of this thermoplastic polymer blend, PC/ABS reinforced with 2% and 3% w of SWCNT. The material parameter values are shown in Table 6.

**Figure 13:**
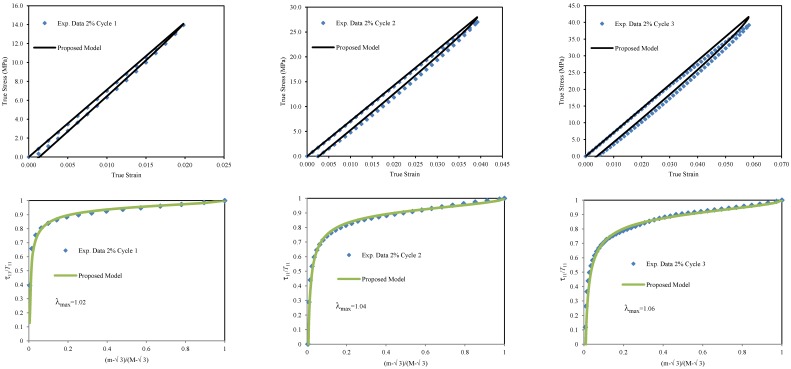
Comparison of theoretical predictions obtained from Equation (25) with experimental data of PC/ABS material reinforced with 2% weight fraction of SWCNT.

The comparison of experimental data and theoretical prediction for a 2% w SWCNT fraction are illustrated in Figure 13 for the first three loading and unloading cycles. It is important to mention that during the tensile test, we loaded and unloaded the samples at stretch values below the material yield point to capture only the material elastic behavior. As we can see from Figure 13, our proposed constitutive material model predicts experimental data well. Also, it is noteworthy that the RMSE value does not exceed 1.1866, as shown in Table 7.

**Table 6:** Entry and fitted parameters values for PC/ABS-SWCNT composite material.

Parameter	2% w SWCNT	3% w SWCNT	Fitting parameter	2% w SWCNT	3% w SWCNT
Volume fraction, *f*	0.0088	0.0031	*µ*_R_ (MPa)	359.4880	346.0830
*N*	200	200	*Δ*	0.7	0.8
*n*	1	1	*A*_1_ (MPa)	1882.3	1960.6
*α*	1/2	1/2	*A*_2_ (MPa)	−4849.9	−47239
*N*	1	1	*C* (MPa)	13	10

**Table 7:** Predicted quadratic (RMSE) and median square (MSE) error values for PC/ABS-reinforced material data shown in Figure 13. Here, “Diff” represents the maximum percentage error between theoretical and experimental data.

2% w SWCNT	Cycle 1		Cycle 2		Cycle 3	
	Loading	Unloading	Loading	Unloading	Loading	Unloading
RMSE	0.1069	0.1655	0.3922	0.6546	0.9776	1.1866
MSE	0.0114	0.0274	0.1538	0.4285	0.9558	1.4081
Diff	1.20%	2.21%	3.74%	3.19%	6.20%	5.44%

Based on the above results, we can conclude that the enhanced material model described by Equation (25) describes the mechanical behavior of polymeric materials reinforced with SWCNT quite well.

## 6. Conclusions

In this paper, we have modified the Cantournet *et al.* model to describe the mechanical behavior of polymeric materials reinforced with carbon nanotubes in which softening and residual strains effects are considered. First, we have used an energy density function derived from the statistical mechanics to predict the mechanical behavior of the MWCNT and SWCNT elastomeric composite materials. Then, we have used a non-monotonous softening function and a residual strain energy density function to characterize elastomeric materials reinforced with carbon nanotubes when subjected to uniaxial extension cyclic loading conditions. It was shown that the assumptions made by Cantournet and co-workers to consider an anisotropic material as an equivalent “isotropized” material provide a simple model that can be used to predict the behavior of MWCNT- and SWCNT-reinforced polymers. The use of this model and the assumption that cyclic load induced non-monotonous material stress-softening behavior give as a results a general constitutive equation that can be used to characterize the stress-softening and permanent set effects in reinforced polymeric materials. We found for the polymeric material BIMSM reinforced with MWCNT that theoretical predictions, when compared to experimental data, do not exceed the root mean square error value of 0.075 for a 12.2% or less of weight fraction concentrations; similarly, we found a RMSE below 0.392 for natural rubber-reinforced with MWCNT [30]; a predicted RMSE value below 0.0401 for a polybutadiene elastomer reinforced with carbon black; and a RMSE of 0.955 for a thermoplastic material reinforced with 2% w of SWCNT fraction with a corresponding maximum error of 6.20%. Moreover, the modified Cantournet *et al.* model described by Equations (19) and (25) requires the determination of only six material parameters to predict experimental data of reinforced polymeric materials, *i.e.*, the determination of the chain number of links, *N*, the macroscopic material parameters *δ* and *μ*_R_, two “isotropized” micromechanics parameters *A*_1_ and *A*_2_, and the phenomenological residual strain parameters *C*. Here, in all cases, we have used the value of *n* = 1 in Equation (25). Finally, we can see that our phenomenological non-monotonous softening function described by the simple constitutive relation (7) herein applied to the BIMSM-MWCNT, NR-MWCNT, PC/ABS-SWCNT and PBR-CB-reinforced elastomers has shown to predict well the stress-softening experimental data for different loading and unloading cycles.
